# Workplace-based skin cancer screening improves self-examination confidence and knowledge in firefighters

**DOI:** 10.1016/j.jdin.2026.05.025

**Published:** 2026-06-13

**Authors:** Moira Shea, Mallory deCampos-Stairiker, Justin Ng, Hannah Zhao, Emile Latour, Elizabeth Stoos, Sancy Leachman, Christine Kannler

**Affiliations:** a School of Medicine, Oregon Health & Science University, Portland, Oregon; b Department of Dermatology, Oregon Health & Science University, Portland, Oregon; c Northeast Dermatology Associates, North Andover, Massachusetts

**Keywords:** education, risk factors, skin cancer, sun protection practices

*To the Editor:* Existing literature indicates firefighters have an increased risk of skin cancer compared to the general population, possibly due to occupational exposures.^1^ Although studies have explored risk factors increasing firefighter risk, there remains a paucity of knowledge about their attitudes, knowledge, and behaviors toward sun safety, which are strong predictors of completing self-skin examinations for early skin cancer detection.^1^^,^^2^ Limited awareness may result in delayed diagnosis in this high-risk group, and we hypothesized that interaction with dermatology specialists through screenings and patient education could improve skin cancer knowledge.

Of over 1,500 firefighters in Boston, Massachusetts, a sample attended skin cancer screenings in 2022 (*n* = 424) and 2023 (*n* = 524). Participants were surveyed on demographics, sun practices, risk factors, and attitudes surrounding skin cancer (Supplementary Methods 2, available via Mendeley at https://doi.org/10.17632/xx99wnmty9.1). Knowledge was assessed via a 10-question quiz. Differences in attitudes, knowledge, and confidence in performing self-skin examinations were compared among firefighters attending 2 versus 1 screening (*P* < .05).

Spot Me forms had an overall response rate of 99.2% across 2 years, while knowledge surveys had a response rate of 92.3%. Most participants were between the age 30 and 59, and 75% lacked a regular dermatologist. By self-report, 16.5% use sunscreen and 29.0% use sun-protective clothing “rarely” or “never.” Regarding risk factors, ≥4 blistering sunburns before age 20 were reported by 29.4%, and 17.1% used tanning beds (Table I). Those who attended 2 screenings were more likely to report confidence with self-skin examinations (*P* = .016) and had higher knowledge quiz scores (*P* = .005) compared to those who attended one.

**Table I:** Patient self-reported sun practices expressed as overall percentages

Sun practices	All subjects	One screening	Two screenings
Ever had skin cancer			
Yes	4.3	4.7	4.2
No	95.7	95.3	95.8
Previous type of skin cancer			
Melanoma	16.2	16.7	16.1
Basal cell carcinoma	29.7	33.3	29.0
Squamous cell carcinoma	21.6	16.7	22.6
Unsure	32.4	33.3	32.3
Has a regular dermatologist			
Yes	24.5	25.9	24.2
No	75.5	74.1	75.8
Uses sunscreen			
Always	23.7	22.1	24.0
Sometimes	59.9	64.0	58.9
Rarely	11.1	7.4	12.0
Never	5.4	6.6	12.0
Wears sun-protective clothing			
Always	10.6	11.3	10.4
Sometimes	60.4	57.9	60.9
Rarely	19.6	21.8	19.1
Never	9.4	9.0	9.5
No. of painful or blistering sunburns before age 20			
0-3	70.5	62.9	72.4
4+	29.4	37.1	27.6

Our cohort was largely similar to existing studies of firefighters, including predominantly White racial distribution toward the middle of previous estimates (74.1% vs 40.4% to 90.8%),^3^^,^^4^ but our cohort was slightly younger (79.8% < 50 years vs 65.2%).^4^ This could be due to variation in demographics in the local population of Boston compared to Florida or national samples of the above-compared studies.^3^^,^^4^ In contrast to previous studies, this study evaluated participant knowledge and self-examination confidence.^3^^,^^4^

Although there was no formal educational component, we saw a significant increase in confidence in performing self-skin examinations and overall higher skin cancer knowledge on the quizzes for repeat attendees (Table II). Although this may reflect increased participation by firefighters with higher baseline knowledge, it may also indicate that learning passively through discussion with dermatology providers during screening is a valid way to improve confidence and knowledge.

**Table II:** Posteducation attitudes surrounding skin cancer detection along with posteducation knowledge quiz results expressed as overall percentages comparing participants who attended 1 versus 2 screenings

How confident are you that you are able to check your skin for signs of skin cancer?	Participants who attended 1 screening	Participants who attended 2 screenings	*P*
Not at all confident or not very confident	40.6	30.3	.016
Fairly confident or very confident	59.3	69.7	
Checking my skin for signs of skin cancer will help me detect skin cancer in its early stages	One screening	Two screenings	*P*

Strongly or somewhat disagree	19.3	14.2	.142
Strongly or somewhat agree	80.7	85.8	
How confident are you that you could get your skin checked by a doctor or health care provider?	One screening	Two screenings	*P*

Not at all confident or not very confident	9.1	10.2	.656
Fairly confident or very confident	91.0	89.8	
Mean knowledge quiz scores	Mean score	CI	*P*

One screening	64.0	62.8, 65.1	.005
Two screenings	67.6	65.3, 69.8	

We believe the significant increase in knowledge among attendees of 2 screenings suggests next steps should include formalization of an educational curriculum to further evaluate knowledge increases and attitude change. Sample size, geographic location, and lack of suspicious lesion identification comparison data between 1- and 2-year attendees are limitations of this study. Sun safety and skin cancer curricula have shown significant improvements in other populations, including high school students,^5^ but less exists in the literature for education for those with workplace exposures. We hope this study provides a foundation for both skin-screening-based education or formal instruction for those at increased risk of melanoma.

## Conflicts of interest

Dr Leachman reports an unrelated conflict of interest of receiving an unrestricted educational grant from Castle Biosciences, and serving on advisory boards for Merck, Orlucent, VeriSkin, and Pathology Watch. Drs Shea, deCampos-Stairiker, Ng, Zhao, Latour, and Kannler and author Stoos have no conflicts of interest to disclose.
